# Identification of Drought Tolerant Rice (*Oryza Sativa* L.) Genotypes with Asian and African Backgrounds

**DOI:** 10.3390/plants12040922

**Published:** 2023-02-17

**Authors:** Cyprien Ndikuryayo, Alexis Ndayiragije, Newton Lwiyiso Kilasi, Paul Kusolwa

**Affiliations:** 1 Department of Crop Science and Horticulture, Sokoine University of Agriculture, Morogoro P.O. Box 3001, Tanzania; 2 International Rice Research Institute (IRRI), Bujumbura P.O. Box 5132, Burundi; 3 Burundi Institute of Agricultural Sciences (ISABU), Avenue de la Cathédrale, Bujumbura P.O. Box 795, Burundi

**Keywords:** soil moisture content, quantitative trait loci, irrigated, yield, improvement, Burundi

## Abstract

Drought is among the major abiotic stresses on rice production that can cause yield losses of up to 100% under severe drought conditions. Neither of the rice varieties currently grown in Burundi can withstand very low and irregular precipitation. This study identified genotypes that have putative quantitative trait loci (QTLs) associated with drought tolerance and determined their performance in the field. Two hundred and fifteen genotypes were grown in the field under both drought and irrigated conditions. Genomic deoxyribonucleic acid (DNA) was extracted from rice leaves for further genotypic screening. The results revealed the presence of the QTLs qDTY12.1, qDTY3.1, qDTY2-2_1, and qDTY1.1 in 90%, 85%, 53%, and 22% of the evaluated genotypes, respectively. The results of the phenotypic evaluation showed a significant yield reduction due to drought stress. Yield components and other agronomic traits were also negatively affected by drought. Genotypes having high yield best linear unbiased predictions (BLUPs) with two or more major QTLs for drought tolerance, including IR 108044-B-B-B-3-B-B, IR 92522-45-3-1-4, and BRRI DHAN 55 are of great interest for breeding programs to improve the drought tolerance of lines or varieties with other preferred traits.

## 1. Introduction

Rice is the principal food grain consumed by more than half of the world’s population [1]. However, rice (*Oryza sativa* L.) production faces biotic and abiotic constraints worldwide [2]. The most common biotic constraints include blast, sheath rot, and brown spot in Burundi [3,4]. Among abiotic constraints, drought stress is the major one in rain-fed ecologies [2]. For numerous soils, at least two weeks without rainfall induces noticeable negative differences in drought sensitivity during the vegetative stage, and at least seven days without rainfall causes severe drought damage during the reproductive stage [5]. Drought can cause yield losses of up to 21% under mild drought, up to 51% under moderate drought, and up to 90.6% in severe cases [6], depending on the grown variety, growth stage, degree, and duration of the stress.

Reduced grain yield is a result of morphological responses such as increases in leaf rolling, stomata closure, and leaf tip drying; molecular responses that include changes in gene expression (up/down regulation of transcripts) and the activation of relevant transcription factors and signaling pathways; and physiological and biochemical responses such as reductions in transpiration, photosynthesis, chlorophyll content, membrane stability, stomatal conductance, and increases in osmoprotectants [7]. Drought stress reduces the performance of rice varieties that are grown worldwide [8,9,10].

In East Africa, rice-growing areas are exposed to severe drought [10]. In Burundi, the major constraints on rice production include inputs, flooding, and drought, which accounted for 41%, 30%, and 29%, respectively [11]. In Burundi, irrigated lowland rice is grown in Moso and mostly in the Imbo region. Most farmers in Imbo do not regularly obtain water for irrigation due to insufficient or destroyed infrastructures. Imbo is generally semiarid with low and irregular rainfall that can reach up to 500 mm per year [12]. Neither of the current rice varieties grown in Burundi can withstand such a complicated rainfall pattern. Thus, screening rice varieties suitable for Burundi becomes a priority using an appropriate approach.

A modified conventional breeding approach was suggested to integrate phenotyping, genotyping, and a strategy to screen many lines among which selection can be made [13]. The approach improved the assessment of plant responses to drought stress.

Three levels of drought stress corresponding to 5% (m^3^/m^3^), 10.6% (m^3^/m^3^), and 16% (m^3^/m^3^) soil moisture content, representing severe, moderate, and low drought, respectively, were used by Singh et al. [14] to assess the response of rice cultivars to early-season drought stress. Different methods of screening rice genotypes for drought tolerance have been used by researchers through classical markers or DNA/molecular markers [15,16]. Depending on the specific objective, each type of marker may present advantages and/or disadvantages. Currently, single nucleotides polymorphic markers (SNPs) are mostly used due to their high frequency, low mutation rates, and high-throughput nature [17]. Molecular markers have been a very useful tool mostly in Asian countries (Philippines, India, Nepal, Malaysia, etc.) where different quantitative trait loci (QTLs) for drought tolerance have been identified [16,18].

Polygenic architectures were reported for many traits under both drought and irrigated environments. Previous studies have demonstrated that conditional neutrality is more common than antagonistic pleiotropy [19]. This provides an explanation as to why rice breeders successfully developed drought tolerant rice lines and varieties without a yield penalty in irrigated environments [20]. Shamsudin et al. [21] found a positive interaction between qDTY2.2 and qDTY12.1 in the developed rice lines through marker assisted breeding.

According to IRRI, QTLs with large effects that are qDTY1.1, qDTY2.2, qDTY3.1, qDTY3.2, and qDTY12.1 may be used to improve rice varieties for grain yield under reproductive-stage drought in lowland areas [18]. Introgression or pyramiding of some of these QTLs was successfully achieved, especially in Asia, where drought-tolerant varieties have been released [18,22].

The International Rice Research Institute (IRRI) released genotypes IR 86781-3-3-1-1 and IR 81412-B-B-82-1 in the Philippines, and IR 82077-B-B-71-1, IR 82589-B-B-84-3, and IR 83388-B-B-108-3 in Malawi, Bangladesh, and Nepal, respectively, for drought tolerance in lowland ecosystems. IR 79913-B-176-B-4 and IR 55423-01 are upland varieties that were released in the Philippines and India, respectively [13]. However, there is no information about the use of these genotypes in rice improvement for drought tolerance. Furthermore, there is no report on the release of drought-tolerant rice varieties in Burundi [8].

Therefore, there is a need to effectively utilize the identified QTLs for drought tolerance in developing drought-tolerant rice lines to meet the preferences of producers in Burundi. To enhance breeding efforts, potentially drought-tolerant genotypes were collected from Asia and Eastern and Southern African countries. This study aimed to identify genotypes that have putative QTLs associated with drought tolerance.

## 2. Results

In the current study, the phenotypic data from a sample of 10 plants and genotypic data from two leaves (two plants) of each of the 215 genotypes were subjected to REML analysis or nonparametric testing. The association between phenotype and genotype, which was assessed through Chi-square test of independence, provided insightful results.

### 2.1. Evolution of Drought Stress Symptoms and Nonparametric Test for Scores

Before the drought stress, the tested plants had no symptoms of leaf rolling and drying. The appearance of leaf rolling symptoms started between two and three weeks while symptoms of leaf drying appeared between three and four weeks after stress initiation. The drought stress was observed at the vegetative and reproductive stage. At maturity, with two cycles of drought stress, it was easy to differentiate between stressed and control plants.

The Kruskal-Wallis nonparametric test showed no significant differences between genotypes for both leaf rolling and leaf drying. Significant differences were detected between genotypes by the Kruskal-Wallis nonparametric test for plant phenotypic acceptability, panicle phenotypic acceptability, seed phenotypic acceptability, panicle exertion, and the severity of brown spot under both drought stress and irrigated conditions. Differences in the incidence of sheath rot were only significant between the evaluated genotypes under irrigated conditions (Table 1).

### 2.2. Restricted Maximum Likelihood Analysis for Yield, Yield Components and Other Agronomic Traits

Linear mixed model analysis revealed highly significant (*p* ≤ 0.001) differences in plant height, number of total tillers, days to 50% flowering, days to maturity, number of panicles per plant, panicle length, number of filled grains per panicle, one thousand grain weight, and grain yield of screened genotypes under drought stress and irrigated conditions. Significant differences (*p* ≤ 0.01) were detected among the tested genotypes for spikelet fertility (Table 2).

The mean yield was 2.71 t/ha, the minimum yield was 0.08 t/ha, and the maximum yield was 5.72 t/ha for screened genotypes under drought stress. The BLUP for yield under drought varied between 1.24 and 3.97 t/ha. For the irrigated experiment, the mean yield was 5.10 t/ha, the minimum yield was 1.58 t/ha, and the maximum yield was 9.12 t/ha. BLUP for yield varied between 2.93 and 7.54 under irrigated conditions. The grand mean of yield reduction was 2.33 t/ha, corresponding to 46.15%. More details on individual genotype performance are provided in the Table 3, Tables S2 and S3 of Supplementary Material.

### 2.3. Association between Phenotypic Data and Targeted QTLs for Drought Tolerance

**Figure 1:**
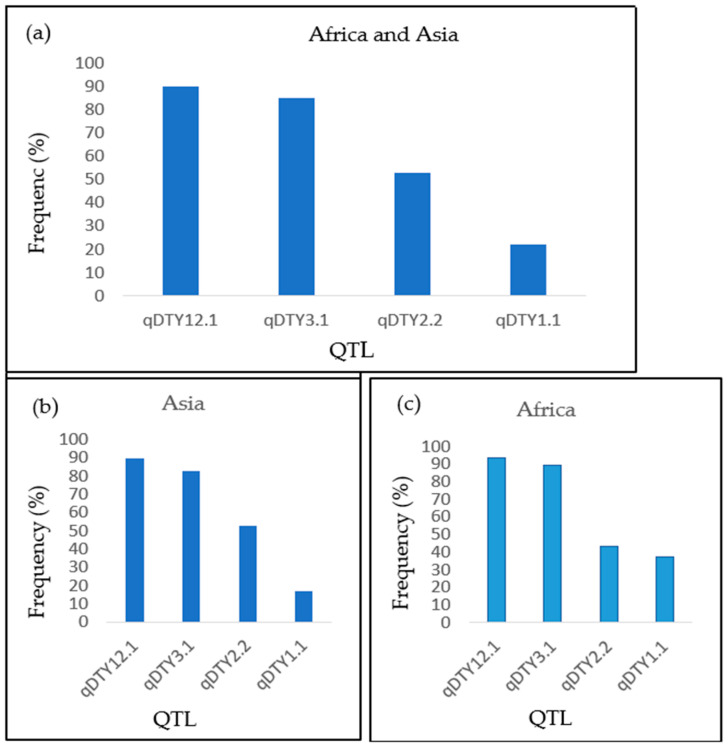
Frequency distribution of targeted QTLs in the evaluated genotypes; (**a**) the percentage of each QTL in the genotypes from both Africa and Asia; (**b**) the occurrence of each QTL in genotypes from Asia alone; (**c**) the frequency of each QTL in genotypes from Africa alone.

Most of the screened genotypes had at least two major quantitative trait loci (QTLs) for drought tolerance. Genotypes that had four, three, two, and one of the targeted QTLs had a mean yield BLUP of 2.38 t/ha, 2.75 t/ha, 2.65 t/ha, and 2.82 t/ha, respectively. Furthermore, the mean stress tolerance index for yield was 0.52, 0.56, 0.53, and 0.56 for genotypes with four, three, two, and one of the targeted QTLs, respectively. The genotypic results showed the presence of the QTLs qDTY12.1, qDTY3.1, qDTY2.2, and qDTY1.1 in 194, 183, 114, and 48 genotypes corresponding to 90%, 85%, 53%, and 22% of the evaluated genotypes, respectively (Figure 1a). The QTL qDTY12.1 was more observed in the genotypes from Asia (Figure 1b) compared to those from Africa (Figure 1c); and the opposite occurred for qDTY1.1.

**Figure 2:**
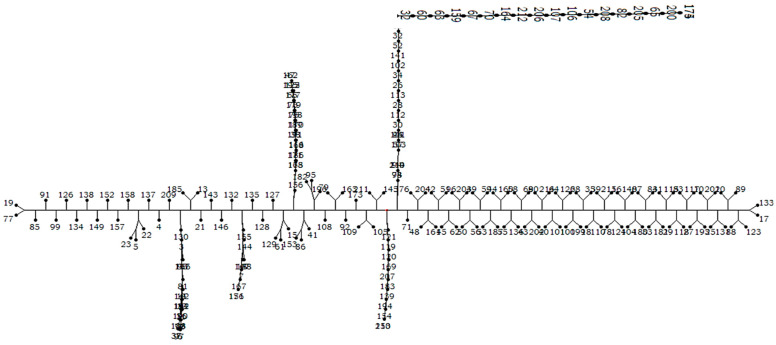
Neighbor-joining tree for evaluated genotypes based on four targeted QTLs.

All the evaluated genotypes formed a total of eight clusters, where the genotypes from Africa and these from Asia shared six clusters and the other two clusters were owned by the genotypes from Asia. The graphical view of the diversity of these genotypes is globally presented in the neighbor-joining tree (Figure 2) and detailed in the dendrogram (Figure A2 of the Appendix A).

The likelihood ratio chi-square showed a significant association between qDTY2.2 and all the phenotypic data under drought stress. The QTL qDTY1.1 was only significantly associated with the yield best linear unbiased predictions under drought stress. Other QTLs were not significantly associated with the phenotypic data (Table 4).

### 2.4. Correlation Analysis for Drought Traits, Yield, and Other Agronomic Traits of Genotypes Evaluated in 2020 at Gihanga Research Station

Correlations between the yield, the number of panicles per plant, and the number of filled grains per panicle were positive and highly significant. Negative and significant correlations were found between yield, leaf rolling, leaf drying, and plant phenotypic acceptability. Strong, positive, and highly significant correlations were detected between the yield, the yield BLUP, and the STI for yield. Correlations between yield and one thousand grain weight, plant height, panicle exertion, and severity of brown spot were negative and non-significant. Strong, positive, and highly significant correlation was detected between the panicle length and the plant height. Highly significant and negative correlations were detected between the plant height, one thousand grain weight, and the number of panicles per plant (Table 5).

## 3. Discussion

This study detected significant differences in leaf rolling and leaf drying among genotypes under drought stress. Furthermore, differences in plant height, number of panicles per plant, one thousand grain weight, and grain yield were significant, suggesting genetic diversity among the tested genotypes. This implies the possibility of selecting most drought-tolerant lines for their further use by farmers or by the breeding program. Comparable results were reported by Mohd Ikmal et al. [23] in BC1 F4 lines. Spikelet fertility and yield components were significantly reduced by drought stress. Similarly, all cultivars subjected to drought stress exhibited a significant grain yield reduction in a study conducted by Adhikari et al. [9]. The effects of drought on morphological and agronomic traits, including leaf area, panicle length, plant height, tillering ability, and efficiency, results in decreased yield [24].

The performance of the evaluated genotypes was better under irrigated conditions than under drought stress conditions. The current results agree with the findings of previous studies on drought where the best cultivars under nonstress conditions exhibited poor performance under stress conditions [9,18]. The reduction in performance of a given genotype increases with drought intensity [14]. This validates the significant efforts that breeders and geneticists have put into coping with water scarcity by finding QTLs and genes for drought tolerance and deploying them in genotypes with different backgrounds [13,18,22]. Therefore, growing drought-tolerant rice varieties is an alternative genetic adaptive strategy to increase rice yield and production in areas where farmers have limited access to water for irrigation [8].

A positive correlation between yield and the number of filled grains per panicle and the number of panicles per hill indicates that a higher number of panicles per plant and a higher number of filled grains per panicle lead to higher yield. Abd Allah et al. [25] reported that the number of panicles per plant, the number of filled grains per panicle, and 100 grain weight are key traits in improving yield under both irrigated and drought stress conditions. The negative correlation between yield and plant phenotypic acceptability is due to the nature of the scale for scoring phenotypic acceptability where higher scores correspond to poor performance [5]. The results imply that the phenotypically desirable genotypes also had higher yields.

Strong, positive, and highly significant correlations between the yield, the yield BLUP, and the STI for yield suggest that high yielding genotypes can be selected based on BLUP or STI. However, the results of this study showed that the correlation coefficient between the yield and the BLUP was greater than the one between yield and STI for yield under drought stress. Furthermore, some genotypes with high STI had low yield under both drought and irrigated conditions (Table 3 and Tables S2 and S3).

Best linear unbiased prediction (BLUP) was reported to be the most efficient prediction method among the commonly used methods for selection [26]. Breeding values imply the ability to perform well in crosses, and they have been recommended to select genotypes with high performance in most of the desirable traits [13].

A significantly negative correlation between the number of days to flowering and the number of filled grains per panicle shows that the longer the cycle of the genotypes, the fewer the number of filled grains per panicle due to the increase in drought intensity at the reproductive stage. Guimarães et al. [27] stated that late-flowering genotypes had high spikelet sterility. The negative and nonsignificant correlation between yield and brown spot shows that yield was slightly affected by this disease. Severe cases of this disease were reported with a yield loss of 50–90% in Bengal [17].

Promising lines were found among screened genotypes during the current study. The genotype IR 108044-B-B-B-3-B-B was classified as the best in the field based on yield BLUP. This genotype has three of the targeted QTLs for drought tolerance, including qDTY2.2, which was significantly associated with all the phenotypic data, and qDTY1.1, which was found in a few genotypes and was significantly associated with the yield BLUPs. In the same way, the genotype IR 92522-45-3-1-4 was ranked fourth based on the yield BLUP from the field where it was under severe drought stress and had three of the targeted QTLs for drought tolerance, including qDTY2.2. Similarly, the genotype BRRI DHAN 55 was under severe drought stress but was ranked seventh in yield BLUP and had three of the major targeted QTLs for drought tolerance, including qDTY1.1. The BLUPs and high yield of these genotypes under drought can enable them to be considered parents for drought tolerance, which can be used to improve existing rice varieties. Dhawan et al. [28] used Nagina 22 as a drought-tolerant parent, for which the yield was 1.77 t/ha under drought stress.

The yield of genotypes IR 97013-8-1-3-2-B, IR 13240-108-2-2-3, WAHIWAHI, and IR 97013-19-1-3-1-B was reduced by rodents that strongly attacked them a few days before pesticide application. The high scores of leaf rolling and leaf drying from the field experiment indicate the presence of a high intensity of drought during this study. High leaf drying induced a reduced yield, confirmed by the negative correlation between these traits. Similar results were obtained by Bocco et al. [24] where more plants with high leaf drying provided lower yields.

The majority of evaluated genotypes had at least two QTLs for drought tolerance providing some yield advantage under drought stress. Appropriate QTL combinations is a good approach for improving drought tolerance [16,21,29]. In the current study, some genotypes with all the four major QTLs were among the worst genotypes under drought stress. Indeed, most of these genotypes with low yield under drought stress were low-yielding even under no stress conditions. Researchers have reported that a high yield potential under no stress is a good indicator of a high yield advantage under drought stress [21]. Another reason could be the interaction between these QTLs, even if conditional neutrality was reported to be more common than antagonistic pleiotropy [19]. The QTLs qDTY2.2 and qDTY3.1 pyramided with qDTY12.1 significantly increased the yield of lines having qDTY12.1 in the study of Shamsudin et al. [21]. Therefore, pyramiding the best combination of alleles with favorable interactions is the best strategy to improve the performance of rice varieties under drought stress [13]. In this study, the QTLs qDTY12.1, qDTY3.1, and qDTY2.2 were present in more than 50% of the evaluated genotypes. Shamsudin et al. [21] found qDTY12.1, qDTY3.1, and qDTY2.2 in 82%, 36%, and 18% of selected pyramided lines, respectively. The analysis of genetic diversity of 60 rice genotypes detected qDTY12.1 and qDTY2.2 in 43.3% and 6.67% of evaluated genotypes, respectively [30].

During the current study, only qDTY2.2 was significantly associated with all the phenotypic data. This indicates that qDTY2.2 is a major QTL for drought tolerance that can be used for the improvement of preferred varieties in Burundi. However, qDTY1.1 was also significantly associated with the yield BLUPs only; further study using other genotypes in Burundi shall help to confirm our findings. Kadam et al. [31] demonstrated the complexity of yield traits under drought stress by detecting very many different QTLs between years and treatments, even by comparing with previous studies using the same genotypes. Overlapping transcriptions between the water-use efficiency and the days to flowering revealed a genetic basis for a trade-off between drought avoidance and drought escape in rice [19]. Therefore, a statistical power analysis accompanying a proper genotypic and phenotypic sampling is very important for QTL studies [32,33]. Drought tolerance is a complex trait that is characterized by low heritability, genotype-by-environment interactions, genetic interactions, and polygenic effects [16]. Furthermore, drought and heat are reported to often occur together. The genes regulating tolerance to these stresses are different but share some signaling pathways [34]. Several genetic management approaches have been suggested by researchers to increase rice production in a changing climate [34,35]. Rice improvement for drought tolerance may continue through pyramiding major QTLs for drought tolerance by crossing elite x elite cultivars or by marker-assisted backcrossing [21,29] involving landraces or wild rice as a source of drought tolerance [36] followed by multi-environmental trials to select for a specific environment or location [16].

## 4. Materials and Methods

### 4.1. Experimental Plant Materials and Study Area

A total of 215 rice genotypes with diverse origins, including potentially aromatic and potentially drought-tolerant genotypes, were screened. Based on information from previous reports, IR 64 was used as a drought susceptible check [37,38] while IR 86781-3-3-1-1 [13] was used as drought tolerant check. These genotypes were provided by the research institutions ISABU and IRRI in accordance with the national and international regulations of plant materials exchange. More details related to the parentage of evaluated genotypes and their geographical origin can be found in the Supplementary Table S1.

**Figure A1:**
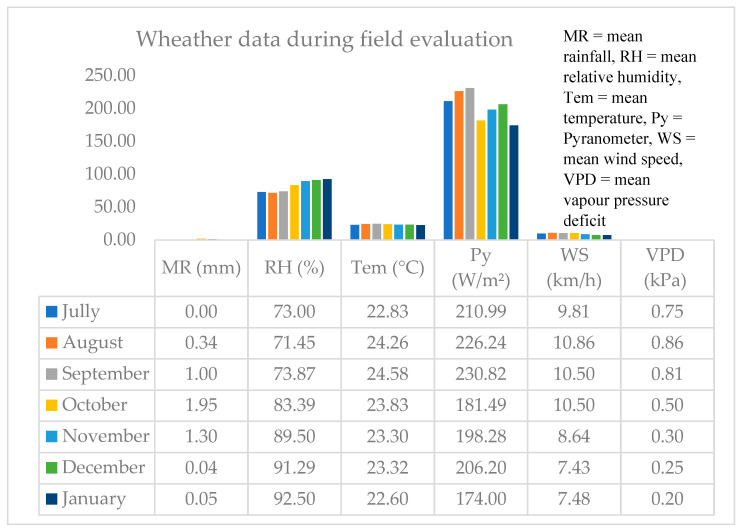
Weather data in the field at Gihanga from mid-July 2020 to early January 2021.

**Figure A2:**
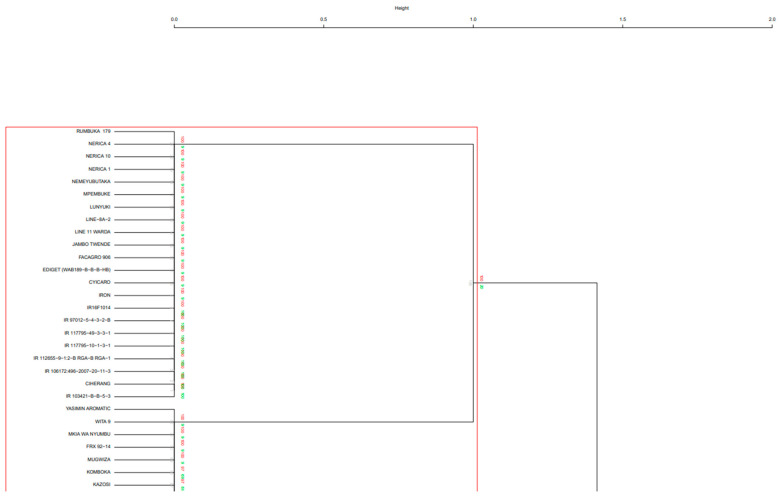

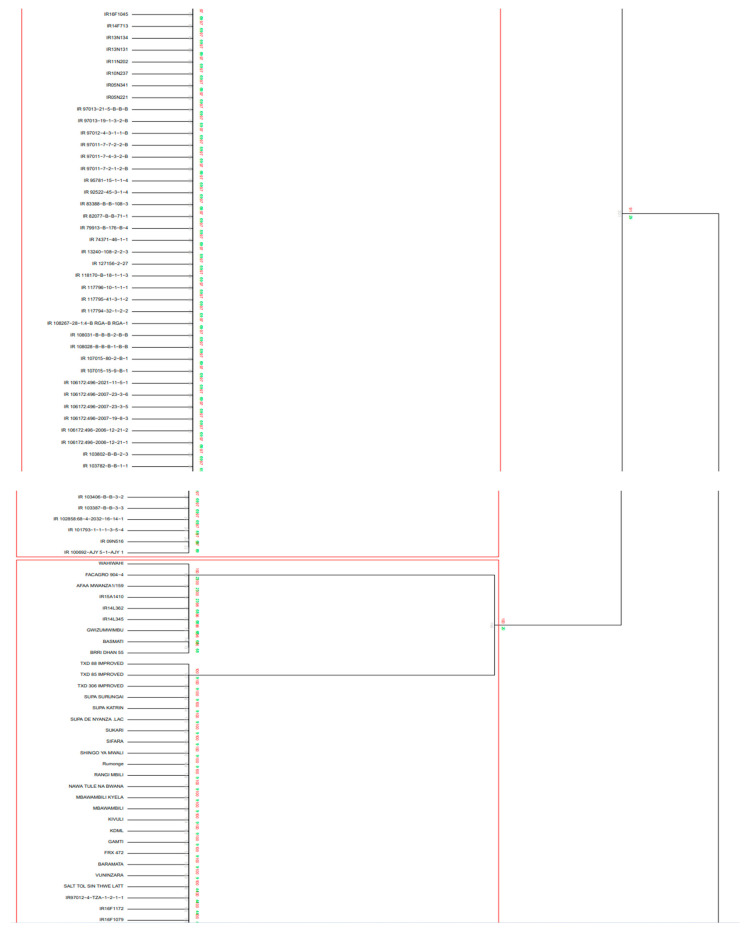

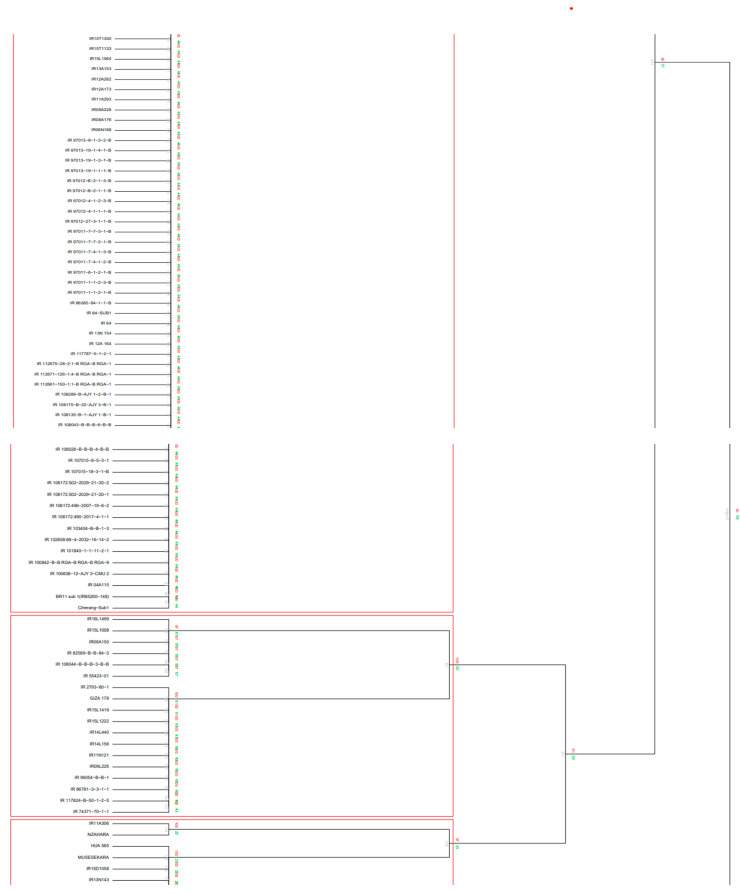

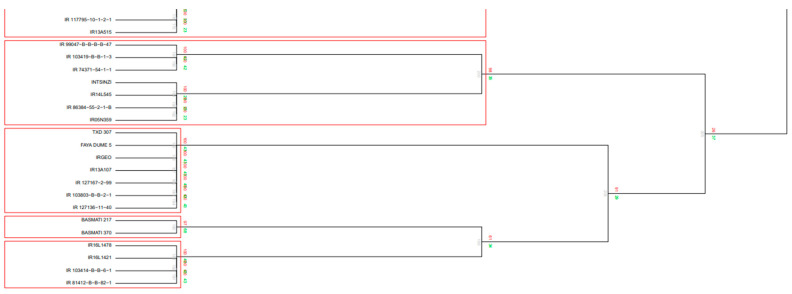
Dendrogram of 215 genotypes based on four targeted QTLs for drought tolerance.

The experiments were set in the field at Gihanga Central Imbo in Burundi, which is located at 29°2′14.3″ E and 3°10′23.9″ S. With an elevation of 839 m above sea level, the annual mean temperature is 24 °C. During this study, the total rainfall was 141.63 mm in five months and half. More information on weather data is provided in the Appendix A (Figure A1).

### 4.2. Field Experimental Design, Agricultural Practices, and Drought Treatment

Tested genotypes were grown in an alpha lattice design with 2 replications for both irrigated and non-irrigated experiments. Seeding was done on 17 July 2019 and transplanting was performed three weeks after seeding. Each genotype had five rows and occupied 5.4 m^2^ with only one seedling per hill. At transplanting time, fertilizers were applied according to the formula NPK 75-30-30 at a rate of 65 kg of DAP, 29 kg of urea, and 50 kg of K_2_O per ha, as recommended by the Ministry of Environment, Agriculture, and Livestock [39]. Drought stress was initiated at 28 days after the last date of transplanting by draining the field of the drought experiment [38]. Other agricultural practices were performed as recommended by the MINEAGRIE [39].

Soil samples were taken from the field before plowing and were analyzed for further field and drought management. The results of soil analysis are presented in the Appendix B (Table A1). Soil classification into texture classes was performed according to Moormann and Van Breemen [40] using Texture AutoLookup (TAL) 42 software. The permanent wilting point was then determined [41]. Two polyvinyl chloride (PVC) pipes measuring 1.2-m-long × 2-inch diameter with small perforations at the bottom were installed 1 m below the soil surface in different replicates of the field for water table measurements.

Another PVC pipe measuring 0.52 m long × 2 inches in diameter was installed 0.5 m below the soil surface. Measurement of the water table was performed using a meter stick from one week after draining the field until harvesting time. Using the Hand Held (HH2, version 4.3) soil moisture meter, the soil moisture content was recorded once the genotypes had started showing symptoms of leaf rolling. Re-irrigation was performed once the soil moisture content was almost at the permanent wilting point. Water was removed from the plots after 6 h to initiate the second cycle of drought stress [38]. Experimental plants underwent two cycles of drought stress.

### 4.3. Genotyping Procedure

Two leaf samples were harvested from each genotype at six weeks after transplanting before booting. These samples were taken to the laboratory at IRRI-Burundi, where they were punched by an EP100 machine and kept in the wells of plates at −80 °C for 24 h. Samples in plates were later transferred to a lyophilizer for 48 h. To check the presence or absence of the major QTLs for drought tolerance, these samples were subjected first to genomic deoxyribonucleic acid (DNA) extraction and then to the Kompetitive Allele Specific Polymerase chain reaction (KASP) method in the INTERTEK laboratory according to the method described by Kanyange [3]. The SNP markers targeting major QTLs for drought tolerance are provided in the Table 6.

### 4.4. Data Collection and Analysis

The drought traits of leaf rolling and leaf drying were recorded at most twice a week once some genotypes showed symptoms and at the end of the stress cycle before reirrigation [38]. The mean leaf rolling scores were obtained using the IRRI standard evaluation system for rice [5], where: 0 = leaves healthy, 1 = leaves start to fold (shallow), 3 = leaves folding (deep V-shape), 5 = leaves fully cupped (U-shape), 7 = leaf margins touching (0-shape), and 9 = leaves tightly rolled.

In the same way, mean leaf drying scores were obtained using the IRRI standard evaluation system for rice [5] where: 0 = no symptoms, 1 = slight tip drying, 3 = tip drying extended up to ¼ length in most leaves, 5 = one-fourth to ½ of all leaves dried, 7 = more than 2/3 of all leaves fully dried, and 9 = all plants apparently dead. The plant height, the number of days to 50% flowering, and the number of days to maturity were recorded.

At the maturity period, data were recorded from ten hills in each plot for the number of tillers per plant, number of panicles per plant, panicle length, number of filled grains per panicle, and 1000 filled grain weight [5]. Data collected from the whole plot included phenotypic acceptability of the plant, phenotypic acceptability of panicle, phenotypic acceptability of seeds, panicle exertion, and grain yield. The filled grains from each plot were weighed using a high-accuracy electronic scale, and grain yield (t/ha, 13%) for each genotype was computed using Formula (1) [42]:

Grain yield (t/ha) = (Plot grain weight (Kg/plot) × 10,000 × (100 − GMC))/((100 − 13) × (harvested plot area) × 1000)(1)

where t/ha is tons per hectare and GMC is grain moisture content (%).

The percentage reduction in grain yield [28] and the stress tolerance index (STI) [9] were calculated using Formulas (2) and (3):

Yield reduction (%) = (Y_ins_ − Y_is_) × 100/Y_ins_ (2)

STI = (Y_ins_ Y_is_)/((Y_ins_)^2^) (3)

where, Y_ins_ is the Yield of ith genotype under non-stress condition and Y_iS_ represents the yield of ith genotype under stress condition. Other data collected from the whole plot included diseases that were present in many plots or that had high severity, such as sheath rot and brown spot.

The collected data was subjected to restricted maximum likelihood (ReML) and mixed linear model analysis using Genstat14. Genotypes were attributed fixed effects, while replicates and blocks had random effects. Means were separated using Tukey’s test at the 5% level of significance [43] after detecting significant differences. Scores for drought traits, phenotypic acceptability, and diseases were subjected to Kruskal–Wallis nonparametric tests [33] using the Statistical Tool for Agricultural Research (STAR).

The genotypic data underwent a numerical scoring method by assigning one to a positive allele and zero to a negative allele. To test the association between phenotypic and genotypic data, a chi-square test of independence was performed using STAR. To display the genetic dissimilarity of tested genotypes, a weighted neighbor-joining tree was constructed in DARwin 6.0.21 [44]. To generate a dendrogram, the genotypic data was subjected to hierarchical clustering with 1000 bootstrap *p*-values in KDCompute 1.5.2.beta [45].

Through multivariate analysis, a biplot was generated and helped to reduce the number of traits to consider for correlation analysis. To determine genotypes that can be considered as potential parents for drought tolerance improvement in rice, best linear unbiased predictions (BLUPs) were calculated using R statistical software.

## 5. Conclusions

The current study demonstrated that drought stress significantly reduced yield for all tested genotypes. The intensity and duration of drought stress may be considered when selecting drought-tolerant rice lines. Genotypes having high yield best linear unbiased predictions (BLUPs) with two or more major QTLs for drought tolerance, including IR 108044-B-B-B-3-B-B, IR 92522-45-3-1-4, and BRRI DHAN 55, are of great interest for drought tolerance improvement in Burundi. However, further studies using other genotypes, including segregating populations, are needed in Burundi to confirm the effectiveness of qDTY 2.2 and qDTY 1.1 in controlling drought tolerance and their interaction with other potentially putative QTLs. This will enable the breeding program to successfully use these putative QTLs for drought tolerance improvement of locally grown varieties in a changing climate. Future research shall provide a list of genes within the QTLs recommended by IRRI for grain yield under drought stress, their interactions, and their mode of action in the light of ABA-mediated drought tolerance pathways [46], ERECTA-mediated drought tolerance [47], and DREB-based ABA-independent drought tolerance responses [48].

## Figures and Tables

**Table 1:** Kruskal-Wallis rank sum test for phenotypic acceptability and diseases cores for field experiments.

Drought Experiment						
**Change**	**PPA**	**PaPA**	**SPA**	**PE**	**ISR**	**BS**
Chi-Square	303.18	278.15	290.15	288.03	244.90	315.80
d.f.	214	214	214	214	214	214
Pr > Chi-Square	0.0001	0.0021	0.0004	0.0005	0.0700	0.0000
Irrigated (control)						
Chi-Square	285.54	324.65	340.54	324.89	280.46	262.60
d.f.	214	214	214	214	214	214
Pr > Chi-Square	0.0008	0.000	0.000	0.000	0.002	0.013

PPA = plant phenotypic acceptability, PaPA = panicle phenotypic acceptability, SPA = seed phenotypic acceptability, PE = panicle exertion, ISR = incidence of sheath rot and BS = severity of brown spot, d.f. = degree of freedom.

**Table 2:** Restricted Maximum Likelihood analysis for yield and other agronomic traits for field experiments.

Drought Experiment											
**Source of Variation**	**d.f.**	**PH**	**TT**	**DFl**	**DM**	**PP**	**PL**	**SF**	**NFGP**	**TGW**	**Yield**
Rep	1	1.48 ns	93.39 *	1771.74 ***	1857.27 ***	75.55 *	26.83 *	23.82 ns	1013.10 ns	1.66 ns	1.48 ns
Rep/block	9	69.99 ***	11.15 ***	71.71 ***	103.36 ***	9.31 ***	4.70 ***	237.77 **	648.80 **	10.63 **	2.93 ***
Genotype ^a^	213/214	222.07 ***	9.85 ***	261.53 ***	225.60 ***	8.83 ***	4.37 ***	170.07 **	586.78 ***	24.42 ***	1.95 ***
Residual ^b^	181.03–187.08	22.21	2.72	18.86	19.44	2.44	1.36	96.42	282.10	4.48	0.77
LEE ^b^	181.03–187.08	24.98	3.10	21.04	21.82	2.79	1.54	106.30	310.46	4.93	0.87
Cv%		6.73	16.56	4.55	3.62	16.93	6.17	28.68	21.05	9.10	34.33
SED		5.00	1.76	4.59	4.67	1.67	1.24	10.31	17.62	2.22	0.93
Irrigated experiment (control)											
Rep	1	251.35 ns	30.13 ns	422.89 *	205.09 ns	30.25 ns	144.07 ***	1096.10 ***	1832.30 ns	55.83 *	1.07 ns
Rep/block	9	143.94 **	18.03 ***	42.41 ***	42.54 ***	14.38 ***	2.52 *	^c^	1038.20 ***	7.40 ns	1.44 ns
Genotype	214	288.90 ***	6.30 ***	131.02 ***	118.35 ***	5.38 ***	2.95 ***	59.56 **	798.65 ***	31.64 ***	2.95 ***
Residual ^b^	185.28–193.01	55.19	2.66	4.96	7.64	2.48	1.10	25.97	254.10	4.17	1.065
LEE ^b^	185.28–193.01	59.69	2.93	5.45	8.35	2.73	1.19	^c^	280.23	4.48	1.1535
CV%		7.96	15.38	2.48	2.34	15.98	4.80	6.08	13.70	7.69	21.083
s.e.d.		7.73	1.71	2.34	2.89	1.65	1.09	5.18	16.74	2.12	1.074

*** Significant at *p* ≤ 0.001, ** significant at *p* ≤ 0.01, * significant at *p* ≤ 0.05, ns = nonsignificant at *p* ≤ 0.05, ^a^ = the degree of freedom of genotype varies because of missing data, ^b^ = the degree of freedom of residual and LEE varies because of the nature of the lattice layout during analysis, LEE = lattice effective error, CV = coefficient of variation, s.e.d. = standard error of difference, d.f. = degree of freedom, PH = plant height, TT = number of total tillers, DFl = days to flowering, DM = days to maturity, PP = number of panicles per plant, PL = panicle length, SF = spikelet fertility, NFGP = number of filled grains per panicle, TGW = one thousand grain weight, ^c^ = analysis was performed according to randomized complete block design because blocks were not significant in alpha lattice design for variable spikelet fertility.

**Table 3:** Summary of means and genotypic information for the ten best and ten worst genotypes and checks based on yield BLUPs under drought stress.

DESIGNATION	YBs	Ys	Yns	RY	STIY	PPAs	PPAns	SM	qDTY12.1	qDTY2.2	qDTY3.1	qDTY1.1
IR 108044-B-B-B-3-B-B	3.97	5.06 ab	6.20	18.27	0.82	1.04	4.11	11.98	+:+	+:+	-:-	+:+
IR 108031-B-B-B-2-B-B	3.91	5.03 ab	6.08	17.30	0.83	4.06	2.36	13.34	+:+	+:+	+:+	-:-
MUSESEKARA	3.73	4.50 abcd	5.34	15.88	0.84	3.03	2.99	15.79	+:+	-:-	-:-	-:-
IR 92522-45-3-1-4	3.64	4.42 abcd	6.76	34.68	0.65	3.99	3.21	10.28	+:+	+:+	+:+	-:-
IR 97011-7-4-1-3-B	3.57	4.42 abcd	5.83	26.80	0.73	1.94	2.35	13.73	+:+	-:-	+:+	-:-
YASIMIN AROMATIC	3.54	4.11 abcd	5.62	26.96	0.73	3.69	3.05	16.59	+:+	+:+	+:+	-:-
BRRI DHAN 55	3.53	4.08 abcd	4.70	13.20	0.87	2.90	3.87	10.65	+:+	-:-	+:+	+:+
IR 103421-B-B-5-3	3.51	3.94 abcd	5.17	23.87	0.76	3.97	2.88	8.47	?	*	+:+	+:-
IR 112671-126-1:4-B RGA-B RGA-1	3.48	4.14 abcd	7.10	41.58	0.58	4.02	3.09	13.06	+:+	-:-	+:+	-:-
BASMATI	3.45	3.96 abcd	6.58	40.62	0.59	3.97	2.95	12.94	+:+	-:-	+:+	+:+
IR 106172:496-2007-23-3-6	1.90	1.44 abcd	4.83	70.15	0.30	7.13	4.92	9.21	+:+	+:+	+:+	-:-
SUPA DE NYANZA–LAC	1.87	1.15 abcd	5.69	79.86	0.20	6.99	5.09	14.02	+:+	-:-	+:+	-:-
JAMBO TWENDE	1.81	1.03 bcd	4.27	75.84	0.24	6.99	7.23	13.96	+:+	+:+	+:+	+:+
IR 107015-18-3-1-B	1.77	0.29 d	2.90	89.99	0.10	9.00	9.14	12.07	+:+	-:-	+:+	-:-
NERICA 10	1.74	1.08 abcd	3.11	65.14	0.35	6.03	6.36	13.83	+:+	+:+	+:+	+:+
EDIGET (WAB189-B-B-B-HB)	1.68	0.95 bcd	2.33	59.41	0.41	5.07	7.33	15.21	+:+	+:+	+:+	+:+
LINE-8A-2	1.68	0.99 abcd	2.62	62.36	0.38	6.01	5.78	14.60	+:+	+:+	+:+	+:+
MKIA WA NYUMBU	1.68	0.81 cd	3.29	75.34	0.25	8.94	6.76	7.79	+:+	+:+	+:+	-:-
NERICA 4	1.60	0.83 cd	2.56	67.48	0.33	5.98	3.62	14.71	+:+	+:+	+:+	+:+
FRX 472	1.24	0.08 cd	4.89	98.43	0.02	9.02	6.41	11.07	+:+	-:-	+:+	-:-
IR 86781-3-3-1-1^+^	3.30	3.74 abcd	5.00	25.22	0.75	3.97	3.80	14.82	+:+	+:+	-:-	-:-
IR 64^+^	3.02	3.45 abcd	6.10	43.50	0.56	8.06	4.18	8.98	+:+	-:-	+:+	-:-

+ = Check; a,b,c,d = means that share the same letter are not significantly different, they belong to the same group; YBs = yield best linear unbiased prediction under drought stress, Ys = yield under drought stress, Yns = yield under non stress conditions, RY = percentage of reduction in yield, STIY = stress tolerance index for yield, PPAs = plant phenotypic acceptability under drought stress, PPAns = plant phenotypic acceptability under irrigated conditions, SM = soil moisture, qDTY = quantitative trait loci for drought tolerance, +:+ = homozygote, +:- = heterozygote, -:- = negative for targeted QTL, ? = absent in one sample and present in the second sample for qDTY 12.1, * = was homozygote in one sample and heterozygote in other samples for qDTY2.2.

**Table 4:** Chi-Square test of independence for phenotypic and genotypic data.

Variable	d.f.^1^	qDTY2.2 LRC	qDTY3.1 LRC	qDTY12.1 LRC	qDTY1.1 LRC
YBs	132	171.90 *	132.64 ns	106.16 ns	161.08 *
Ys	214	297.83 ***	191.04 ns	141.97 ns	228.33 ns
DFl	213	295.05 ***	188.26 ns	139.20 ns	228.33 ns
DM	213	295.05 ***	191.04 ns	141.97 ns	228.33 ns
SF	214	297.83 ***	191.04 ns	141.97 ns	228.33 ns
TT	210	296.37 ***	190.68 ns	141.75 ns	225.31 ns
PH	213	296.37 ***	190.68 ns	141.75 ns	225.31 ns
PP	213	296.37 ***	190.68 ns	141.75 ns	225.31 ns
PL	209	293.60 ***	187.91 ns	138.98 ns	225.31 ns
TGW	214	297.83 ***	191.04 ns	141.97 ns	228.33 ns
NFGP	214	297.83 ***	191.04 ns	141.97 ns	228.33 ns
PPA	176	252.05 ***	161.22 ns	107.29 ns	183.60 ns
PaPA	168	242.01 ***	159.49 ns	116.65 ns	180.15 ns
SPA	169	234.74 ***	162.26 ns	113.20 ns	195.06 ns
PE	172	234.37 **	155.67 ns	124.29 ns	199.56 ns
LD1	109	146.27 **	109.16 ns	88.19 ns	131.91 ns
LD2	143	195.55 **	141.44 ns	105.56 ns	168.01 ns
LD3	168	241.33 ***	140.76 ns	110.43 ns	179.10 ns
LD4	177	259.69 ***	147.35 ns	114.92 ns	195.06 ns
LR1	137	192.73 **	141.44 ns	103.47 ns	159.33 ns
LR2	175	236.46 **	166.76 ns	130.88 ns	194.69 ns
LR3	172	234.74 ***	158.44 ns	122.56 ns	191.24 ns
LR4	179	250.69 ***	172.31 ns	133.65 ns	187.42 ns
BS	197	278.42 ***	185.49 ns	130.88 ns	217.24 ns
ISR	193	260.74 ***	174.40 ns	124.29 ns	214.47 ns

^1^ = the degrees of freedom vary because of variation in cells with expected frequency, YBs = yield best linear unbiased prediction under drought stress, Ys = yield under drought stress, DFl = days to flowering, DM = days to maturity, SF = spikelet fertility, TT = number of total tillers, PH = plant height, PP = number of panicles per plant, PL = panicle length, TGW = one thousand grain weight, NFGP = number of filled grains per panicle, PPA = plant phenotypic acceptability, PaPA = panicle phenotypic acceptability, SPA = seed phenotypic acceptability, PE = panicle exertion, LD = leaf drying, LR = leaf rolling, BS = severity of brown spot, ISR = incidence of sheath rot, d.f. = degree of freedom, qDTY = quantitative trait loci for drought tolerance, LRC = likelihood ratio Chi-square, *** Significant at *p* ≤ 0.001, ** significant at *p* ≤ 0.01, * significant at *p* ≤ 0.05, ns = nonsignificant at *p* ≤ 0.05.

**Table 5:** Correlations between traits of evaluated rice genotypes for the field drought experiment.

	YIELD	DFl	LD1	LD4	LR4	NFGP	TGW	PH	PP	PPA	BS	STIY
DFl	0.08 ns											
LD1	−0.20 **	0.07 ns										
LD4	−0.15 *	0.12 ns	0.32 ***									
LR4	−0.20 **	0.10 ns	0.18 *	0.50 ***								
NFGP	0.41 ***	−0.26 ***	−0.03 ns	−0.04 ns	−0.18 **							
TGW	−0.11 ns	−0.20 **	0.07 ns	−0.09 ns	−0.41 ***	−0.05 ns						
PH	−0.11 ns	−0.07 ns	0.12 ns	−0.03 ns	−0.21 **	0.18 **	0.40 ***					
PP	0.43 ***	0.51 ***	−0.04 ns	0.02 ns	0.18 **	−0.19 **	−0.48 ***	−0.51 ***				
PPA	−0.57 ***	0.05 ns	0.12 ns	0.19 **	0.30 ***	−0.24 ***	−0.07 ns	−0.09 ns	−0.12 ns			
BS	−0.07 ns	−0.17 *	0.06 ns	0.12 ns	0.14 *	−0.07 ns	−0.10 ns	−0.11 ns	0.05 ns	0.30 ***		
STIY	0.75 ***	0.15 *	0.21 **	0.28 ***	0.19 **	0.36 ***	0.05 ns	0.01 ns	0.20 **	0.48 ***	0.03 ns	
YdB	0.95 ***	0.10 ns	0.22 **	0.19 **	0.22 **	0.38 ***	0.15 *	0.11 ns	0.44 ***	0.57 ***	0.09 ns	0.72 ***

*** Significant at *p* ≤ 0.001, ** significant at *p* ≤ 0.01, * significant at *p* ≤ 0.05, ns nonsignificant at *p* ≤ 0.05, DFl = days to flowering, LD = leaf drying, LR = leaf rolling, NFGP = number of filled grains per panicle, TGW = one thousand grain weight, PH = plant height, PP = number of panicles per plant, PPA = plant phenotypic acceptability, BS = severity of brown spot, STIY = stress tolerance index for yield, YdB = yield best linear unbiased prediction.

**Table 6:** SNP markers and targeted QTLs for drought tolerance.

SNP ID	QTL	Favorable Allele	Unfavorable Allele
snpOS0085	qDTY3.1	A	G
snpOS0091	qDTY12.1	T	C
snpOS00400	qDTY1.1	G	C
snpOS00412	qDTY2.2	C	A

SNP = single nucleotide polymorphisms, QTL = quantitative trait loci.

## Data Availability

The data supporting the conclusions of this article are provided within the article, in the appendices and in the Supplementary Materials. If more information such as row data is needed, it will be provided by the corresponding author on reasonable request.

## Supplementary Materials

The following supporting information can be downloaded at: https://www.mdpi.com/article/10.3390/plants12040922/s1, Table S1: Origin of genotypes, Table S2: Means for agronomic traits including yield and yield components, drought traits and diseases under drought stress in the field. Table S3: Means for agronomic traits including yield and yield components and diseases under irrigated conditions in the field.

## Appendix B

**Table A1:** Results of soil analysis for Gihanga site in Imbo Lowland.

Soil Texture						
Rep	Block	Sand (%)	Silt (%)	Clay (%)	Texture	PWP (%)
1	1	85.8	5.81	12.41	loamy sand	8.5
1	2 and 3	62.25	22.54	13.41	sandy loam	9.1
1	4 and 5	81.91	5.4	11.5	loamy sand	8.5
1	6 and 7	84.36	5.94	7.06	loamy sand	8.5
1	8 and 9	83.14	5.67	6.19	loamy sand	8.5
2	1 and 2	83.28	4.85	10.35	loamy sand	8.5
2	3 and 4	79.42	5.91	11.76	sandy loam	9.1
2	5 and 6	76.74	5.3	15.02	sandy loam	9.1
2	7 and 8	74.2	4.62	14.04	sandy loam	9.1
2	9	79.19	4.97	12.83	sandy loam	9.1
pH and some nutrients content						
pH H_2_O	% N	P (mg/kg)	K(mEq/100 g)	Na(mEq/100 g)	Zn(mg/kg)	Fe(mg/kg)
5.5	0.09	32.4	0.33	0.14	0.37	443

PWP = permanent wilting point, pH = hydrogen ion concentration, N = nitrogen, P = phosphorus, K = potassium, Na = sodium, Zn = zinc, Fe = iron, % = percent, mg = milligram, kg = kilogram, mEq = milliequivalent.
